# Cost-effectiveness of surgical treatment of thumb carpometacarpal joint arthritis: a value of information study

**DOI:** 10.1186/s12962-023-00438-8

**Published:** 2023-05-01

**Authors:** Alfred P. Yoon, David W. Hutton, Kevin C. Chung

**Affiliations:** 1 Section of Plastic Surgery, Department of Surgery, University of Michigan Medical School, 2130 Taubman Center, 1500 East Medical Center Drive, Ann Arbor, Ann Arbor, MI, Michigan 48109-0340 USA; 2 Health Management and Policy, University of Michigan School of Public Health, Ann Arbor, MI USA; 3 Section of Plastic Surgery, Department of Surgery, University of Michigan Medical School, 2130 Taubman Center, 1500 East Medical Center Drive, Ann Arbor, Ann Arbor, MI, Michigan 48109-0340 USA

**Keywords:** Thumb carpometacarpal joint arthritis, CMC arthritis, Basal joint arthritis, Cost-effectiveness analysis, Value of information analysis, Expected value of perfect information

## Abstract

**Background:**

Thumb carpometacarpal (CMC) joint arthritis is one of the most prevalent arthritic conditions commonly treated with trapeziectomy alone or trapeziectomy with ligament reconstruction and tendon interposition (LRTI). We evaluate the cost-effectiveness and value of perfect and sample information of trapeziectomy alone, LRTI, and non-operative treatment.

**Methods:**

A societal perspective decision tree was modeled. To understand the value of future research in comparing quality-of-life after trapeziectomy, LRTI, and non-operative management we characterized uncertainty by fitting distributions to EQ-5D utility data published from the United Kingdom hand surgery registry. We used Monte Carlo simulation for the probabilistic sensitivity analysis and to evaluate the value of perfect and sample information.

**Results:**

Both trapeziectomy alone and LRTI were cost-effective compared to non-operative management ($2,540 and $3,511/QALY respectively). Trapeziectomy alone (base case total cost $8,251, QALY 14.08) was dominant compared to LRTI (base case total cost $8,798, QALY 13.34). However, probabilistic sensitivity analysis suggested there is a 12.5% chance LRTI may be preferred at a willingness-to-pay of $50,000/QALY. Sensitivity analysis revealed postoperative utilities are the most influential factors in determining cost-effectiveness. The value of perfect information was approximately $1,503/person. A study evaluating the quality-of-life of 1,000 patients in each arm undergoing trapeziectomy alone or LRTI could provide an expected $1,117 of information value. With approximately 40,000 CMC arthroplasties performed each year in the U.S., the annual value is close to $45 million.

**Conclusions:**

Trapeziectomy without LRTI appears to be the most cost-effective procedure in treating late-stage CMC arthritis and should be considered as first-line surgical treatment. There is substantial societal value in conducting additional research to better understand the relative quality-of-life improvements gained from these two common hand surgeries.

**Supplementary Information:**

The online version contains supplementary material available at 10.1186/s12962-023-00438-8.

## Introduction

Carpometacarpal (CMC) arthritis of the thumb is the second most common degenerative condition of the hand [1] with a prevalence of 15% in adults older than 30 [2, 3]. Its prevalence increases with age, as one study found that CMC arthritis has a 90% prevalence in patients older than 80 [4]. Approximately 8% of men and 25% of women older than 50 are afflicted with CMC arthritis and up to 20% of these patients require treatment [5–7]. CMC arthritis results in progressive joint tenderness, stiffness, and weakness that impairs hand function. Most mild cases of CMC arthritis are managed conservatively with splints, physical therapy, and corticosteroid injections. However, as the disease worsens patients elect to undergo surgery to mitigate pain. Various surgical techniques for treating CMC arthritis have been described in the literature; however, the most common approaches in the United States are trapeziectomy with tendon interposition ligament reconstruction (LRTI) followed by trapeziectomy alone [8, 9].

Prior studies demonstrated similar outcomes after trapeziectomy and trapeziectomy with LRTI [10–13]. Nonetheless, trapeziectomy with LRTI is the preferred treatment for most surgeons in the United States [8, 9]. Although some experts suggest trapeziectomy with LRTI may have higher complication rates than trapeziectomy alone [14], most studies have not found significant differences in complication rates between the two procedures [10, 12, 13, 15]. A randomized prospective study comparing trapeziectomy alone to trapeziectomy with LRTI revealed no significant differences in pain relief, postoperative function, and thumb strength [12]. A 2017 Cochrane review of studies comparing trapeziectomy to LRTI concluded that most available evidence were of low quality [16].

Cost-effectiveness analysis may be helpful to policymakers as trapeziectomy with LRTI may be more expensive and have uncertain health benefits. To date, no cost-effectiveness study has compared the economic impact of choosing one procedure over the other and the value of further studies on this topic is unclear. Given that the evidence comparing the two procedures are insufficient, we aimed to contribute to the literature and assist decision-makers by providing an economic analysis perspective. Value of information (VOI) analysis is a systematic strategy to assess whether the cost of acquiring additional information via research to reduce decision uncertainty in cost-effectiveness analyses is worth the potential benefit [17]. All cost-effectiveness analysis results have decision uncertainty based on the quality of available evidence; however, this decision uncertainty can lead to costly consequences for the healthcare system. A VOI analysis weighs the benefit of being able to make a better policy decision using the information gained from additional research against the cost of conducting that additional research [17].

We asked whether trapeziectomy or trapeziectomy with LRTI is more cost-effective in treating late-stage CMC arthritis. We also informed the value of future studies and the information needed to decrease the uncertainty of our cost-effectiveness findings.

## Methods

### Data source

A comprehensive literature review was conducted to identify complication rates after trapeziectomy and trapeziectomy with LRTI including unresolved pain, tendon rupture, and complex regional pain syndrome (CRPS) [10–13, 15, 18–23]. All retrospective and prospective studies comparing the interventions were included. This study adhered to the Consolidated Health Economic Evaluation Reporting Standards (CHEERS) reporting guideline. According to the University of Michigan institutional review board, this study fell under the University of Michigan’s policy for research using publicly available data. Under this policy and in accordance with federal regulation for human research, IRB approval was not required.

### Model design

**Fig. 1:**
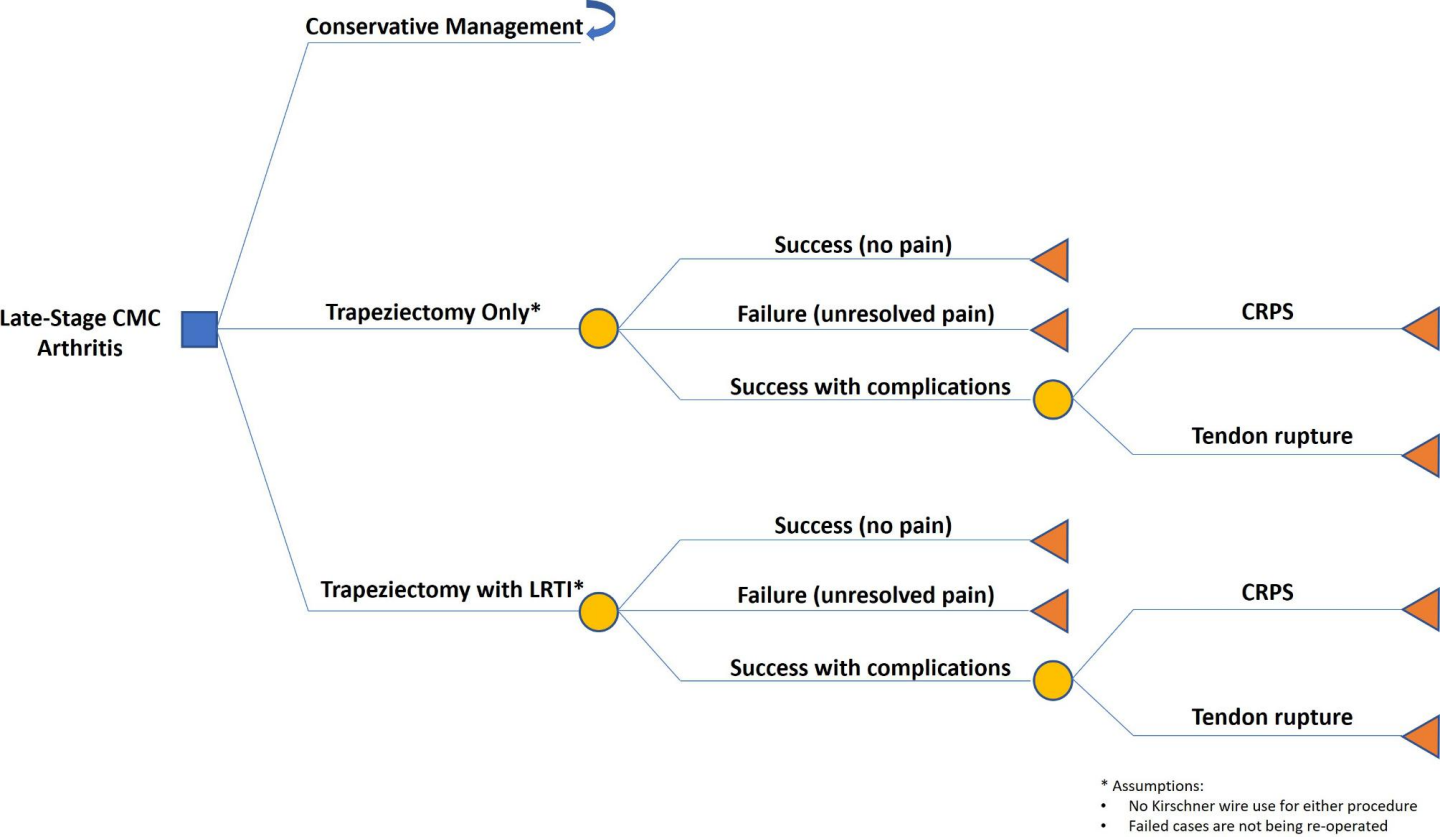
Decision tree model. It was assumed that no hardware was used for either trapeziectomy only or trapeziectomy with LRTI

Before conducting a value of information (VOI) analysis, a model-based cost-effectiveness analysis was conducted to compare trapeziectomy alone, trapeziectomy with LRTI, and non-operative management. The model was built using a societal perspective in the context of United States healthcare. A decision tree was built with three treatment arms for end-stage CMC arthritis: conservative management, trapeziectomy, and trapeziectomy with LRTI. Respective complications over a lifetime horizon were considered given that CMC arthritis is a life-long condition (Fig. 1). The base case scenario was a 62-year-old patient who has been managing CMC arthritis conservatively for an extended time with bracing and corticosteroid injections considering surgical intervention for pain relief. Three scenarios were compared: conservative management where patients decide against intervention and continues to live with pain, trapeziectomy only, and trapeziectomy with LRTI. For patients who chose surgical interventions, they incurred costs from the initial procedure, rehabilitation, and recovery (Table 1). Postoperative time off work [24] and complication rates [10, 12, 13, 15, 19, 22] were determined from the average reported values in the literature. Only complications that require surgical intervention or result in long-standing suffering in quality of life, such as unresolved pain, CRPS, and tendon rupture, were considered in the model because it was assumed that minor complications are temporary and do not incur consequential cost or affect health utilities. Average treatment specific complication rates reported in the literature are shown in Table 1. We calculated the overall value of information with a time horizon of 10 years to estimate a conservative value of information in an elderly patient population with limited years of healthy life remaining. We conservatively estimated 40,000 CMC arthroplasties per year which was extrapolated from the New York State Ambulatory Surgery and Services Database [25]. In 2014, there were 2,630 total CMC arthroplasties performed in New York according to the New York State Ambulatory Surgery and Services Database. Because all CMC arthroplasties are outpatient procedures, we decided this was the most appropriate database to estimate prevalence. At the time, the state of New York had a population of 19.7 million and the U.S. had a population of 318 million. Assuming that New York’s prevalence of CMC arthroplasty is similar to the national mean, we estimated the yearly total CMC arthroplasties performed in the U.S. at approximately 40,000 cases. We then calculated the value of sample information based on the utility increase with trapeziectomy and the utility increase with LRTI examining trial sample sizes between 250 and 1,250 patients. A systematic review of U.S. randomized controlled trials found that the trial cost per patient varied widely from $43 to $103,254 per patient with a median cost of $17,020 per patient [26]. Because the trials included in this study were large cardiovascular and oncology trials, we conservatively set the average cost for a study participant in a trial to $10,000. We assumed all physicians will convert from performing one procedure to another considering the new information provided by the clinical trial (perfect implementation).

**Table 1:** List of Model Variables for Base Case Scenario

	Base	Low	High	Source	Distribution
**Population**					
Age at time of injury	62	61	64	Thorkildsen and Rokkum(28)	Uniform
Life Expectancy	86	82	98	Center for Disease Control and Prevention (CDC)(27)	Uniform, but 1 year above age at time of injury
**Complication Rates**					
Conservative Management					
Unresolved pain (failure)	1	0.95	1		N/A
Trapeziectomy					
CRPS^†^	0.029	0.016	0.095	Davis, Brady, and Dias(13); Salem and Davis(20)	Beta
Tendon rupture	0	0	0		N/A
Unresolved pain (failure)	0.079	0.04	0.13	De Smet et al.(17)	Beta
LRTI^‡^					
CRPS^†^	0.04	0.018	0.08	Davis, Brady, Barton, Lunn, and Burke(15); Gangopadhyay et al(12)	Beta
Tendon rupture	0.043	0.043	0.043	Belcher and Nicholl(10)	Beta
Unresolved pain (failure)	0.066	0.01	0.13	De Smet et al.(17)	Beta
**Costs**					
Direct costs					
Conservative Management	0	0	0		
Trapeziectomy (CPT 25,210)					
Physician Fee	508.39	448.62	702.02	2020 cm National Physician Fee Schedule(29) (national payment amount base, min MAC, max MAC)	Truncated Normal (> 0)
Anesthesia Fees	151	135.9	166.1	cm(29)	Truncated Normal (> 0)
Facility Fees	2830.4	2547.36	3113.44	Medicare and HCUP (2020 OPPS)(23)	Truncated Normal (> 0)
Hand Therapy Cost	477.28	432.36	673.75	Indiana Hand Center(22),2020 cm National Physician Fee Schedule(29) (national payment amount base, min MAC, max MAC)	Truncated Normal (> 0)
LRTI^‡^ (CPT 25,447)					
Physician Fee	854.53	756.87	1181.55	2020 cm National Physician Fee Schedule(29) (national payment amount base, min MAC, max MAC)	Truncated Normal (> 0)
Anesthesia Fees	194	174.6	213.4	cm(29)	Truncated Normal (> 0)
Facility Fees	2830.4	2547.36	3113.44	Medicare and HCUP (2020 OPPS)(23, 29)	Truncated Normal (> 0)
Hand Therapy Cost	477.28	432.36	673.75	Indiana Hand Center(22), 2020 cm National Physician Fee Schedule(29) (national payment amount base, min MAC, max MAC)	Truncated Normal (> 0)
Complications (Cost)					
CRPS^†^					
Physician Fee	444	399.6	488.4	2020 cm National Physician Fee Schedule(29) (national payment amount base, min MAC, max MAC)	Truncated Normal (> 0)
Tendon repair (CPT 26,356)					
Physician Fee	822.05	739.845	904.255	2020 cm National Physician Fee Schedule(29) (national payment amount base, min MAC, max MAC)	Truncated Normal (> 0)
Anesthesia Fee	124.7233	112.251	137.1957	cm(29)	Truncated Normal (> 0)
Hospital Fee	2623.34	2361.006	2885.674	Medicare and HCUP (2020 OPPS)(23, 29)	Truncated Normal (> 0)
Wage Calculations					
Average annual mean	51,960	10,000	250,000	Bureau of Labor and Statistics(30)	Truncated Normal (> 0)
Time off for recovery and rehab (days)					
Conservative Management	0	0	0	Indiana Hand Center(22)	N/A
Trapeziectomy	30	14	84	Indiana Hand Center(22)	Truncated Normal (> 0)
LRTI^‡^	30	14	84	Indiana Hand Center(22)	Truncated Normal (> 0)
Retirement age	66	60	70	Social Security(44)	Truncated Normal (> 0)
Discount Rate	0.03	0	0.07	N/A	N/A
**Utilities**					
Baseline					
Conservative Management	0.675	0.26	0.8	Lane et al.(25)	Normal
Relative increases for trapeziectomy and LRTI^‡^					
Trapeziectomy Increase	0.16	0.14	0.19	Lane et al.(25)	Normal
LRTI^‡^ Increase	0.14	0.11	0.17	Lane et al.(25)	Normal

†CRPS: Complex Regional Pain Syndrome

‡LRTI: Ligament Reconstruction and Tendon Interposition

CMS: Center for Medicare and Medicaid Services

All distributions are parameterized with means equal to the base case and standard deviations of ¼ the range between the low and high. If a distribution is listed as N/A, the parameter is not varied in the probabilistic sensitivity analysis or value of information analysis

### Health states

Health utilities after non-operative management, trapeziectomy only, and LRTI were based on a retrospective study of a prospectively collected Euroqol 5 Dimension (EQ-5D) indices at baseline, 3, 6, and 12 months postoperatively in 1,456 patients and quantified the change in EQ-5D index from before to after surgery [27]. Utilities at 12 months were assumed to remain constant throughout the remainder of life. The utility increase reported from the U.K. Hand Registry for trapeziectomy and trapeziectomy with LRTI were used to calculate postoperative utilities using data fitting and bootstrapping to estimate the confidence intervals of the mean utility increases from the procedures (Supplemental Content). A long-term decrement in utility was applied to patients who contract complex regional pain syndrome according to prior studies who made similar assumptions while studying musculoskeletal conditions [28]. Decrement in utility after failure was also derived from the study by Lane et al. Both average life expectancy and remaining years of life were derived from the U.S. Centers for Disease Control and Prevention life Tables [29]. Because thumb CMC arthritis generally affects older patients, the age range in the model was varied between 61 and 64 [30].

### Costs

The model included the following direct costs: physician fees, anesthesia fees, facility fees, hand therapy costs, and additional costs incurred by complications. Indirect costs included patients’ wages lost from recovery. Physician and facility fees were determined using Medicare reimbursement rates from Centers for Medicare Services [31]. Current Procedural Terminology (CPT) codes 25,210 and 25,447 were used to value trapeziectomy and trapeziectomy with LRTI, respectively. Anesthesia costs for the initial procedure and surgical complications were calculated using 2021 Medicare reimbursement and anesthesia conversion factors. Hand therapy costs were based on hand therapy guidelines from the Indiana Hand Center using the CPT codes listed in Supplemental Table 1 [24]. Patient wages lost from recovery time were accounted for as indirect costs. The lengths of time off work were also based on the recommendations provided by the Indiana Hand Center guidelines [24]. Base case scenario assumed 30 days off work for both procedure types. Because CMC arthritis is a prevalent condition without preference of one type of workforce over another, the 2018 Bureau of Labor Statistics national mean salary was used as the base case salary [32]. We assumed that all patients returned to full-time duty without restrictions after recovery. A societal perspective analysis was taken based on recommendations from previous literature [33].

### Statistical analysis

#### Cost-effectiveness analysis

We first conducted a cost-effectiveness analysis between trapeziectomy and trapeziectomy with LRTI. The incremental cost-effectiveness ratio (ICER) is calculated by dividing the difference in cost between two interventions by the difference in their effect. The primary analysis was conducted using the base case parameters denoted in Table 1. We then performed sensitivity analyses varying key model parameters to determine the most influential factors. All direct costs were varied by 10% from the Medicare amount. The uncertainty of utilities was varied by a standard deviation based on the Gaussian distribution of the EQ-5D differences between pre and postoperative patients who underwent trapeziectomy or trapeziectomy with LRTI [27]. One-way sensitivity analysis was conducted to identify parameters that most influence the outcomes. A willingness to pay threshold of $100,000 per quality-adjusted life year was used as a definition of cost-effectiveness [34, 35] Excel Office 365 (Microsoft Corp., Redmond, Wash.) was used for modeling and analysis.

#### Value of information analysis

The primary outcome of this study was expected value of sample information ($/person), which is the amount a decision maker is willing to pay to reduce uncertainty in a decision. Because value of information analysis is based on Bayesian decision theory, probabilistic decision modeling is required [36–38]. Therefore, we conducted a probabilistic sensitivity analysis using Monte Carlo simulation to quantify the level of uncertainty in the results from the overall uncertainty from model inputs [39]. We used expected value of perfect partial information for all parameters to determine which were most important for sampling information. We also conducted an analysis of the expected net benefit of sampling that included costs of operating the trial, but not opportunity costs of the trial. When varying the numbers of individuals in the trial, we assumed equal numbers of persons in each arm of the trial.

## Results

### Model-based CEA results

The main difference in the direct cost between trapeziectomy and trapeziectomy with LRTI stemmed from the difference in physician reimbursement fee and anesthesia time. For the base case scenario, LRTI had the highest total cost of $8,798 whereas the cost of trapeziectomy was slightly lower at $8,251. Conservative management was modeled to have $0 total expenditure because the frequency of clinic follow-up was assumed to be equivalent to postoperative clinic follow-up visits after either procedure. The average utility increase after trapeziectomy and trapeziectomy with LRTI based on the study by Lane et al. were similar (0.16 vs. 0.14, respectively). Corresponding lifetime quality-adjusted life years (QALYs) were higher for the trapeziectomy group (14.14) compared to the LRTI group (13.80), and the conservative management group (11.43). Compared to conservative management, both trapeziectomy and LRTI were cost-effective treatments with ICER of $3,045/QALY and $3,711/QALY respectively. When comparing trapeziectomy to LRTI, LRTI was dominated by trapeziectomy because trapeziectomy cost less compared to LRTI but accrued more QALYs.

### Sensitivity analysis

Literature reported long-term complication rates of either procedure were relatively low at 3 to 6%, not significantly affecting the overall results. Exploration of data with one-way sensitivity analyses revealed that utility was the most important parameter that determined cost-effectiveness (Supplemental Table 2). The uncertainties in utilities of both procedures were high (Table 1). If the 12-month utility of LRTI was just 0.01 greater than that of trapeziectomy, LRTI became a cost-effective treatment compared to trapeziectomy with an ICER of $3,233/QALY (Supplemental Fig. 1). Probabilistic sensitivity analysis revealed that at a willingness to pay threshold of $100,000 per QALY, there is an 87, 13, and 0% chance that trapeziectomy, LRTI, and conservative management being cost-effective with the current knowledge of outcomes and their uncertainties.

### Estimated value of information results

**Fig. 2:**
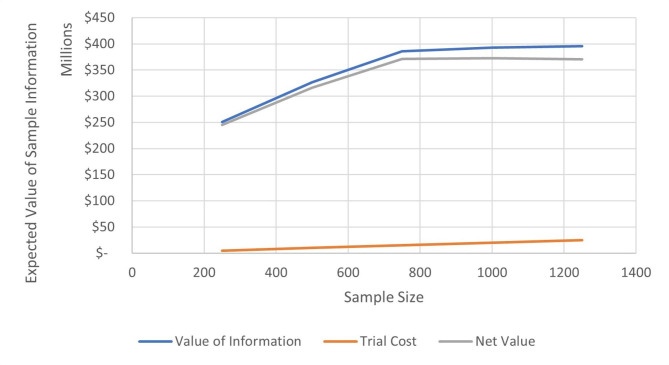
Value of information analysis results. Assuming a prevalence of 40,000 CMC arthroplasties per year in the United States, a clinical trial with 250 patients yielded an estimated value of information of $713 per person with a net value of $245 million over 10 years. This net value of information reached its maximum at a sample size of 1,000 patients

The expected value of perfect information was $1,503 per person. In other words, a healthcare decision maker (surgeon, payer, state or federal government, societies making clinical guidelines) is willing to pay $1,503 per patient to eliminate all uncertainties from the current cost-effectiveness model when choosing between trapeziectomy and trapeziectomy with LRTI. It did not vary significantly when the willingness-to-pay was varied between $50,000 and $200,000 per QALY gained. The expected value of perfect partial information showed that the main uncertainty driving results was the uncertainty in utility gains for trapeziectomy and trapeziectomy with LRTI (Supplemental Table 2). Furthermore, additional sensitivity analysis assuming a negative correlation between complications and utilities potentially increased the value of information by 10% (Supplemental Fig. 2). The expected net benefit of sampling was evaluated for clinical trial sizes from 250 to 1,250 patients in each arm. A clinical trial with 250 patients yielded an estimated value of sample information of $713 per person with an expected net benefit of sampling of $245 million over 10 years. The expected net benefit of sampling plateaued at a clinical trial sample size of 1,000 patients at $373 million dollars (Fig. 2).

## Discussion

Based on the current evidence, trapeziectomy appears to be more cost-effective when compared to trapeziectomy with LRTI. Health utility was the most important parameter that determined cost-effectiveness but there was substantial uncertainty around health utilities. Value of information analysis assessing the net worth of conducting additional research to narrow the uncertainty yielded a net value of $245 million over 10 years.

To our knowledge, no cost-effectiveness study has compared trapeziectomy to trapeziectomy with LRTI for advanced stage thumb CMC arthritis. Two studies investigated health utility changes before and after CMC arthroplasty. Efanov et al. surveyed 32 patients who underwent trapeziectomy with LRTI and determined the postoperative utility to be 0.84 from a time trade-off questionnaire and 0.75 from a standard gamble method [40]. Lane et al. used a prospectively collected EQ-5D index from the U.K. Hand Registry and calculated a 0.16 and 0.14 increase in utilities after trapeziectomy (0.836, 95% CI [0.810–0.859]) and trapeziectomy with LRTI ((0.793, 95% CI [0.784–0.844]) respectively [27]. Our study used the utilities determined by Lane et al. because it was based on a larger patient sample and a prospectively collected validated questionnaire. Neither study investigated the cost-effectiveness or value of information between trapeziectomy and trapeziectomy with LRTI.

Value of information analysis can provide a notion of how valuable it would be to gather additional information on uncertainties relevant to the choice between two interventions. In this study, we found that the utility gains from trapeziectomy and trapeziectomy with LRTI are key drivers of not only cost-effectiveness, but also of the value of information. Complete elimination of uncertainty in a cost-effectiveness result can only be achieved with an infinitely large sample size study that targets all model parameters. The expected benefit of reducing these uncertainties with additional observations (e.g. clinical studies) can be estimated by the expected value of sample information (EVSI). Previous studies implemented value of information analysis to guide clinical studies [41–43]. Randomized clinical trials are the most robust study design to eliminate potential biases such as selection bias or voluntary response bias, albeit more expensive than a prospective study. It is known that when patients are involved in the decision-making process of treatment selection, patient-reported outcomes can be higher which can inflate utility values [44]. To minimize these biases, we chose to model our study with the assumption of conducting a randomized clinical trial with blinding of the patients with regards to which operation, trapeziectomy only or LRTI, they will undergo. Both trapeziectomy only and LRTI can be performed through similar incisions so the patients can be effectively blinded. And given that there is clinical equipoise between the two procedures, such blinding is ethically permissible. Whereas estimating the exact cost and EVSI for a trial that collects just utility versus one that collects additional information such as complications and long-term postoperative outcomes is difficult to estimate, our model’s assumption of $10,000 per participant is a conservative estimate that should be above the true cost of conducting a comprehensive clinical trial. Studies comparing different CMC arthroplasty techniques thus far have been low quality precluding practice-changing conclusions [16]. Given the prevalence of CMC arthroplasty and clinical equipoise among techniques, there is a unique opportunity to conduct a sufficiently powered and well-designed multicenter randomized clinical trial that can yield long-term healthcare savings while maximizing patient outcomes.

### Limitations

The results of this study should be interpreted within the context of several limitations. The utilities used for this study were derived from the U.K. Hand Registry, which may have some differences when compared to the preferences of residents in the U.S. However, given that CMC arthritis generally affects older patients near retirement and both the U.S. and U.K. are developed countries with similar lifestyles, we assumed the utilities are translatable between the two countries. In addition, the utility data comes from a registry, not a randomized trial, so there could be systematic biases. Also, despite our assumption that declines in postoperative utility from long-term complications were captured in the mean utility values from a population registry, the utility decrement from long-term complications is still unclear. Indubitably, the healthcare cost in the U.S. is different when compared to other countries; therefore, our conclusions may not be generalizable to other countries with different healthcare costs. In addition, this is a model that represents the average cohort result within the U.S. healthcare system, which may not be generalizable to patients from other countries. The value of information results are likely an overestimate of the actual societal gain since real-world implementation following publication of study results is likely to be imperfect. Previous studies have demonstrated that imperfect implementation can largely impact the value of information findings [45]. The prevalence of CMC arthroplasty was extrapolated from a statewide Medicare database, which may have over or underestimated its true prevalence. Moreover, the model structure is a decision tree that assumes the health outcomes after one year persist for the remainder of the lifetime. To the extent these improvements in health improve or attenuate over time, this analysis may under- or overestimate the quality-of-life benefits from surgery. Lastly, other more recent techniques involving partial trapeziectomy with or without implant or interposition suspensionplasty were not analyzed in this study and could potentially be cost-effective alternatives [46].

## Conclusion

Based on current data, trapeziectomy appears to be the more cost-effective surgical intervention for CMC arthritis compared to trapeziectomy with LRTI; however, this result is highly contingent on postoperative utilities which have substantial uncertainty. Future studies should contrast postoperative utilities between patients with long-term complications after CMC arthroplasty to those without to ascertain the effects of complications on utilities. Clinical trials narrowing the uncertainties in postoperative utilities of trapeziectomy and trapeziectomy with LRTI can result in a net value of $200 to $300 million in 10 years outweighing the cost of conducting the study. These results suggest that randomized clinical trials comparing trapeziectomy and trapeziectomy with LRTI will likely result in net societal benefit.

## Electronic supplementary material

Below is the link to the electronic supplementary material.

Supplementary Material 1

## Data Availability

The datasets used and/or analysed during the current study are available from the corresponding author on reasonable request.

## List of Abbreviations

CMC: Carpometacarpal
LRTI: Ligament Reconstruction Tendon Interposition
CRPS: Complex regional pain syndrome
VOI: Value of information
EQ-5D: Euroqol 5 Dimension
QALY: Quality adjusted life years
CPT: Current procedural terminology
ICER: Incremental cost-effectiveness ratio
EVSI: Expected value of sample information
