# Supplementary material for: Cost-effectiveness of surgical treatment of thumb carpometacarpal joint arthritis: a value of information study
Source: Cost Eff Resour Alloc. 2023 May 1;21:28. doi: 10.1186/s12962-023-00438-8 (PMC10150507; doi:10.1186/s12962-023-00438-8)
Supplement: Supplementary file 1 — Supplementary Material 1 [file 12962_2023_438_MOESM1_ESM.docx]

**Appendix**

| **Supplemental Table 1. Hand Therapy Current Procedural Terminology (CPT) Codes** | |
| --- | --- |
| Codes | Short Description |
| 97165 | Initial evaluation |
| 97760 | Orthosis fit and train |
| 97140 | Edema control and scar management education |
| 97110 | Therapeutic exercise |
| 97763 | Orthosis re-fit |
| 97010 | Heat therapy |
| 97018 | Paraffin treatment |

**Supplemental Table 2: One-Way Sensitivity Analysis**

|  | **ICER Low** | **ICER High** |
| --- | --- | --- |
| **Population** | | |
| Age at time of injury | Dominated | Dominated |
| Life Expectancy (CDC) | Dominated | Dominated |
| **Complication Rates** | | |
| Conservative Management | | |
| unresolved pain (failure) | Dominated | Dominated |
| Trapeziectomy | | |
| CRPS | Dominated | Dominated |
| tendon rupture | Dominated | Dominated |
| unresolved pain (failure) | Dominated | Dominated |
| LRTI |  |  |
| CRPS | Dominated | Dominated |
| tendon rupture | Dominated | Dominated |
| unresolved pain (failure) | Dominated | Dominated |
| **Costs** | | |
| Direct costs | | |
| Conservative Management | N/A | N/A |
| Trapeziectomy (CPT 25210) | | |
| Physician Fee (including 60 min anesthesia) | Dominated | Dominated |
| Anesthesia Fees (Time units + base units) * anesthesia conversion factor) | Dominated | Dominated |
| Facility Fees | Dominated | Dominated |
| Hand Therapy Cost | Dominated | Dominated |
| LRTI (CPT 25447) | | |
| Physician Fee (including 60 min anesthesia) | Dominated | Dominated |
| Anesthesia Fees (assume 30 more min?) | Dominated | Dominated |
| Facility Fees | Dominated | Dominated |
| Hand Therapy Cost | Dominated | Dominated |
| Complications (Cost) | | |
| CRPS | | |
| Physician Fee | Dominated | Dominated |
| Tendon repair (CPT 26356) | | |
| Physician Fee | Dominated | Dominated |
| Anesthesia Fee | Dominated | Dominated |
| Hospital Fee | Dominated | Dominated |
| Wage Calculations | | |
| Average annual mean | Dominated | Dominated |
| Time off for recovery and rehab (days) | | |
| Conservative Management | Dominated | Dominated |
| Trapeziectomy | Dominated | 21,079.01* |
| LRTI | 5,108.07* | Dominated |
| Retirement age | Dominated | Dominated |
| Discount Rate | Dominated | Dominated |
| **Utilities** | | |
| Baseline | | |
| Conservative Management | Dominated | Dominated |
| relative increases for trapeziectomy and LRTI | | |
| Trapeziectomy Increase | ∞ | Dominated |
| LRTI Increase | Dominated | $3,233.08 |

*****LRTI is less expensive, but has fewer QALYs than trapeziectomy alone. This is the cost-effectiveness of trapeziectomy versus LRTI, so a lower ratio is more favorable to trapeziectomy and a higher ratio would be more favorable to LRTI

**Supplemental Table 3:** **Expected Value of Perfect Partial Information**

|  |  |
| --- | --- |
| **Population** | |
| Age at time of injury | 0 |
| Life Expectancy (CDC) | 0 |
| **Complication Rates** | |
| Conservative Management | |
| unresolved pain (failure) | 0 |
| Trapeziectomy | |
| CRPS | 0 |
| tendon rupture | 0 |
| unresolved pain (failure) | 0 |
| LRTI |  |
| CRPS | 0 |
| tendon rupture | 0 |
| unresolved pain (failure) | 0 |
| **Costs** | |
| Direct costs | |
| Conservative Management | 0 |
| Trapeziectomy (CPT 25210) | |
| Physician Fee (including 60 min anesthesia) | 0 |
| Anesthesia Fees (Time units + base units) * anesthesia conversion factor) | 0 |
| Facility Fees | 0 |
| Hand Therapy Cost | 0 |
| LRTI (CPT 25447) | |
| Physician Fee (including 60 min anesthesia) | 0 |
| Anesthesia Fees (assume 30 more min?) | 0 |
| Facility Fees | 0 |
| Hand Therapy Cost | 0 |
| Complications (Cost) | |
| CRPS | |
| Physician Fee | 0 |
| Tendon repair (CPT 26356) | |
| Physician Fee | 0 |
| Anesthesia Fee | 0 |
| Hospital Fee | 0 |
| Wage Calculations | |
| Average annual mean | 0 |
| Time off for recovery and rehab (days) | |
| Conservative Management | 0 |
| Trapeziectomy | 0 |
| LRTI | 0 |
| Retirement age | 0 |
| Discount Rate | 0 |
| **Utilities** | |
| Baseline | |
| Conservative Management | 0 |
| relative increases for trapeziectomy and LRTI | |
| Trapeziectomy Increase | $240 |
| LRTI Increase | $502 |

**Supplemental Figure 1: Sensitivity on Utility Increase with LRTI**

**Details deriving utilities**

We started with the mean and interquartile range (IQR) of the increase in individual-level EQ-5D utility from the U.K. Hand Registry study[[1](#_ENREF_1)] (Table 2 from that study) from baseline to one year. We then fit a beta distribution (with an offset) to the mean and IQR data reported using Microsoft Excel’s Solver to pick the alpha, beta, and offset parameters to best fit that mean and IQR. This was done separately for both trapeziectomy alone and trapeziectomy with LRTI. This gives us an estimate of individual-level variation in utilities.

**Supplemental Table 4: Estimation of Individual Utility Variation**

|  | Trapeziectomy | Trapeziectomy with LRTI |
| --- | --- | --- |
| Alpha | 0.759767 | 0.251128 |
| Beta | 0.242159 | 0.075076 |
| Offset | -0.59155 | -0.62988 |
| Standard Deviation | 0.303 | 0.366 |

We then used bootstrapping to simulate samples of hypothetical patients with these distributions of individual utility increases to characterize the uncertainty in the population mean utility increases. We simulated groups of 310 and 291 simulated "patients" (the numbers of patients completing patient-reported outcome measures at one year post-operatively) for trapeziectomy and trapeziectomy with LRTI, respectively. A mean is then calculated for each, and then these groups of patients are then simulated again in 400 repetitions. We then had 400 potential "means" that might come from this data. The 10th smallest value is the 2.5th percentile. The 10th largest value is the 97.5th percentile. This is how we estimated the confidence interval of the mean.

**Value of Information Calculations**

We calculated the expected value of complete perfect information, expected value of partial perfect information on each parameter, as well as the expected value of sample information on the two parameters of utility increase with trapeziectomy and the utility increase with LRTI. We found these were the parameters most influential on the results in both one-way sensitivity analysis and in the expected value of perfect partial information analysis (Supplemental Table 3). These parameters were the ones with substantial remaining uncertainty.

We followed the approach from Ades and Claxton[[2](#_ENREF_2)] to calculate the Expected Value of Sample Information. The decision tree is linear in the parameters, so we were able to use simpler algorithms. We ran 10,000 simulations of each sample studies of size, 250, 500, 750, 1000, and 1250 (in each arm). We did not evaluate other sample sizes. We based the priors based on the previous mean utility increases (reported in Table 1) and standard deviations based on the sample sizes from the UK registry.

When simulating the results of hypothetical trials, in each iteration, we draw a “true” population utility increase from the distribution of the population mean effects. Then, we simulate a hypothetical sample mean that we might get from a trial based on that “true” mean, the individual standard deviation from the section “Details deriving utilities” above, and the specified sample size. This new mean of the trial, being an average of individuals is represented as a normal distribution. We then calculate the posterior estimate of what the utility increase would be based on the original sample size from the U.K. Hand Registry and the sample size from this new hypothetical trial using exact formulas based on conjugacy.

With those posterior utility increases, we determined the optimal policy decision for each simulation run. For each of these 10,000 simulations for a specific sample size, we calculated the expected value with and without that sample information to calculate the value of sample information for that particular sample size.

**Supplemental Figure 2: Additional Value of Information with Negatively Correlated Utilities and Adverse Event Rates:**

References

[1] Lane JCE, Rodrigues JN, Furniss D, Burn E, Poulter R, Gardiner MD. Basal thumb osteoarthritis surgery improves health state utility irrespective of technique: a study of UK Hand Registry data. J Hand Surg Eur. 2020; 45(5):436-42.

[2] Ades AE, Lu G, Claxton K. Expected value of sample information calculations in medical decision modeling. Med Decis Making. 2004; 24(2):207-27.
